# Myocardial Infarction As the Initial Presentation of Rituximab-Induced Interstitial Lung Disease: A Case Report

**DOI:** 10.7759/cureus.28179

**Published:** 2022-08-19

**Authors:** Mohamed Mahmoud, Arshan A Khan, Khadija El Kortbi, Hayoung Wang, Joseph Wang

**Affiliations:** 1 Internal Medicine, University of Texas Health Science Center at San Antonio, San Antonio, USA; 2 Internal Medicine, Ascension Saint John Hospital, Grosse Pointe, USA; 3 General Practice, Hassan II University, Casablanca, MAR; 4 Medicine, University of the Incarnate Word School of Osteopathic Medicine, San Antonio, USA

**Keywords:** ground-glass opacities, acute hypoxemic respiratory failure, chemotherapy-related toxicity, lymphoma, interstitial pneumonia, stenotrophomonas maltophilia, pneumocystis jiroveci pneumonia, non-st elevated myocardial infarction, drug induced interstitial lung diseases, rituximab therapy

## Abstract

Rituximab has been widely used alone or in combination therapy to treat B-cell non-Hodgkin lymphoma, chronic lymphocytic leukemia, and various autoimmune diseases. Although it is a relatively safe drug, rare rituximab-induced interstitial lung disease (RTX-ILD) has been reported and can be potentially fatal.

Here, we report a patient with stage 4 mantle cell lymphoma on rituximab who presented with non-ST segment elevation myocardial infarction in the setting of severe respiratory distress. He underwent left heart catheterization that revealed no new obstructive lesions and patent grafts. Extensive Infectious and autoimmune workup was negative except for *Stenotrophomonas maltophilia* pneumonia. The patient was diagnosed later with probable RTX-ILD after exclusion of other etiologies, and he did not show any signs of clinical improvement despite antibiotics and steroid therapy. The patient was then discharged to a long-term acute care hospital, where he eventually passed away.

## Introduction

Rituximab (RTX) is a chimeric monoclonal antibody against CD20 surface antigen, which is widely expressed on the surface of B cells and is used alone or with other chemotherapeutic agents to treat B-Cell non-Hodgkin lymphoma (NHL), chronic lymphocytic leukemia (CLL), and certain autoimmune diseases [1]. It is a relatively safe drug; however, pulmonary toxicity has been increasingly reported in the last two decades. Although a rare side effect, it can be potentially fatal [1,2]. The reported occurrence rate is 0.01-0.03%, but this is likely underestimated due to the widely increasing use of RTX, with some studies reporting incidence rates ranging from 3.7% to 10% [2]. Here we report a case of a probable fatal RTX-induced interstitial lung disease (RTX-ILD) after exclusion of other etiologies in a patient with stage 4 mantle cell lymphoma who presented with non-ST segment elevation myocardial infarction (NSTEMI) in the setting of severe respiratory distress to raise awareness about this potentially lethal complication of RTX therapy.

## Case presentation

A 73-year-old man with stage 4 mantle cell lymphoma on active chemotherapy (bendamustine + RTX), coronary artery disease (CAD) status post coronary artery bypass graft (CABG) for three-vessel disease three years ago to the current presentation, hypertension, and chronic kidney disease presented to the emergency department (ED) with worsening chest pain and shortness of breath for three days. The patient reported constant pressure-like, non-radiating retrosternal chest pain relieved with sublingual nitroglycerin given in the ED. He endorsed chronic intermittently productive cough associated with seasonal allergies, which had not significantly changed, and denied fever, chills, palpitations, or orthopnea. He stated that he finished six cycles of RTX + bendamustine, and the last one was three weeks prior to the presentation.

**Figure 1:**
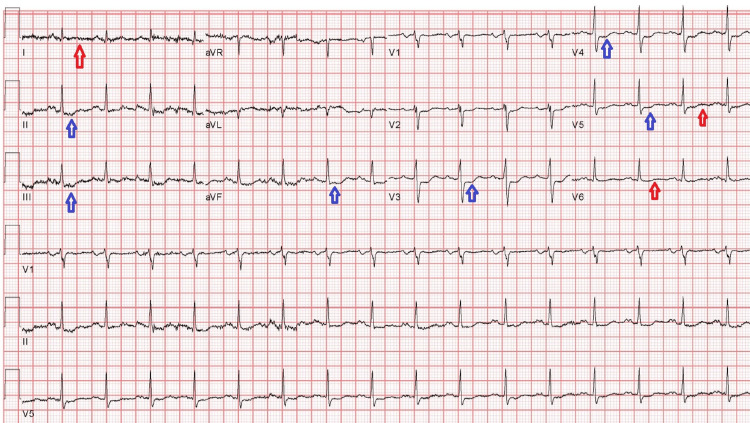
An electrocardiogram (ECG) on presentation ECG showing sinus tachycardia, ST depression in leads II, III, aVF, and V3-V5 (blue arrows), and non-specific T-wave changes (red arrows), suggestive of possible inferolateral myocardial infarction.

In the ED, vital signs were notable with a temperature of 36.8 C, blood pressure of 129/72 mmHg, heart rate of 90 beats per minute, respiratory rate of 24 breaths per minute, and oxygen saturation of 71% on room air, corrected by high flow nasal cannula (HFNC) with FIO2 of 50% and a flow rate of 45L/min. The physical exam was remarkable for the patient being in respiratory distress, tachycardia, and bibasilar crackles. No jugular venous distension or lower extremity edema was noted. An electrocardiogram (ECG) revealed sinus tachycardia, ST depression in leads II, III, aVF, and V3-V5, and non-specific T-wave changes suggestive of possible inferolateral myocardial infarction (Figure 1). Initial laboratory work values are shown in Table 1.

**Table 1:** Initial laboratory values ALT: alanine aminotransferase, AST: aspartate aminotransferase, BNP: B-type natriuretic peptide, INR: international normalized ratio, PCO2: the partial pressure of carbon dioxide, PCR: polymerase chain reaction, * indicates an abnormal value

Lab	Result	Reference range & units
White blood cell count	9.8*	(3.40-10.40) K/mcL
Neutrophils %	90*	(44-75) %
Lymphocytes %	3*	(16-44) %
Hemoglobin	9.6*	Male: (12.8-17.1) g/dL
Hematocrit	28*	Male: (38.6-52.1) %
Platelets	194	(140-377) K/mcL
Sodium	135	(135-145) mmol/L
Potassium	3.9	(3.5-5.1) mmol/L
Chloride	98	(94-106) mmol/L
CO_2_	25	(21-31) mmol/L
Blood Urea Nitrogen	15	(7-25) mg/dL
Creatinine	0.80	Male: (0.60-1.30) mg/dL
Glucose	105*	(60-100) mg/dL
AST	76*	Male: < 35 U/L
ALT	22	Male: < 46 U/L
Total Bilirubin	0.8	(0.2-1.2) mg/dL
Alkaline Phosphatase	93	(45-117) U/L
Total Protein	6.6	(6.2-8.1) g/dL
Albumin	3.3	(3.2-5) g/dL
INR	1.4*	(0.8-1.2)
PH (venous blood gas)	7.36	7.31-7.40
PCO2 (venous blood gas)	45*	(30-40) mmHg
Procalcitonin	1.27*	<0.5 ng/mL
Lactic acid	1.6	(0.5-2) mmol/L
Ammonia	22	(11-32) mcmol/L
Troponin I	5.38*	<0.051 ng/mL
BNP	316*	<100 pg/mL
Blood culture x2	No growth	Negative
COVID-19 PCR	Negative	Negative

**Figure 2:**
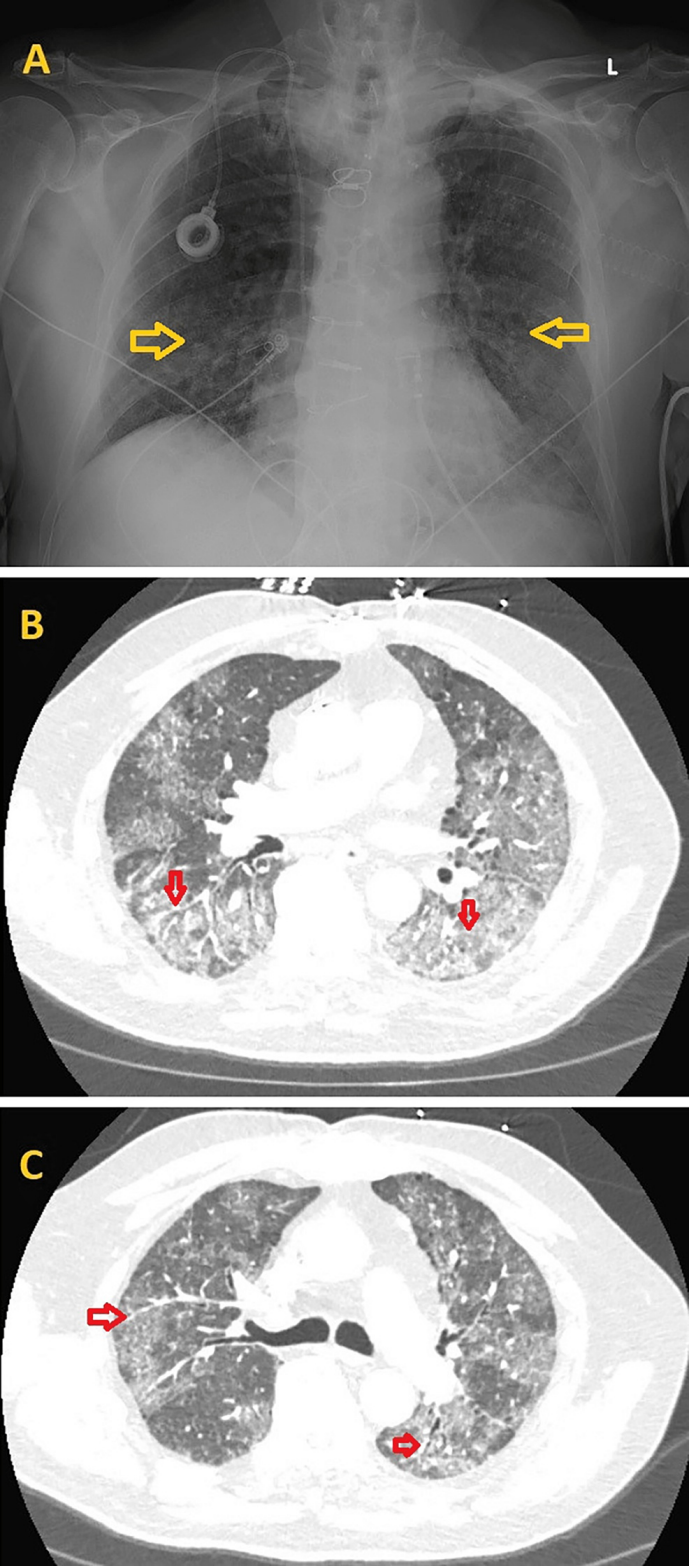
Initial chest X-ray (CXR) and computed tomography pulmonary angiogram (CTPA) findings 2A: CXR showing the prominence of interstitial markings (yellow arrows) predominantly in the mid and lower lung zones, suggestive of pulmonary edema or pneumonitis. 2B, 2C: CTPA showing bilateral ground glass airspace opacities with septal thickening (red arrows)

Chest X-ray (CXR) showed prominence of interstitial markings, suggestive of pulmonary edema or pneumonitis (Figure 2A). The patient was given aspirin 325 mg orally and started on continuous heparin infusion as a part of the acute coronary syndrome (ACS) protocol. He also received intravenous Lasix 40 mg once for concern of flash pulmonary edema. A computed tomography pulmonary angiogram (CTPA) showed bilateral ground glass airspace opacities with septal thickening and no evidence of pulmonary embolus (Figures 2B, 2C).

**Figure 3:**
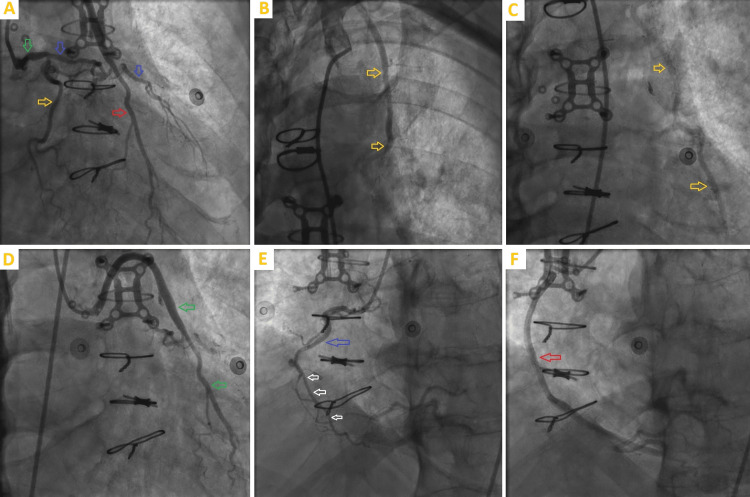
Cardiac catheterization images 3A depicts a left coronary angiogram showing LMCA with mid 50% occlusion (green arrow), LAD (blue arrows) with mid-CTO, patent circumflex (yellow arrow), and patent SVG to the second diagonal artery graft (red arrow). 3B and 3C show patent LIMA to LAD graft. 3D demonstrates patent SVG to the second diagonal artery graft (green arrow). 3E shows RCA (blue arrow) with mid-subtotal occlusion (white arrows). 3F demonstrates patent SVG to rPDA graft CTO: chronic total occlusion, LAD: left anterior descending artery, LIMA: left internal mammary artery, LMCA: left main coronary artery, RCA: right coronary artery, rPDA: right posterior descending artery, SVG: saphenous venous graft

The patient underwent coronary angiography, which revealed multivessel disease with no new obstructive lesions, patent coronary arteries bypass grafts, and left ventricular end-diastolic pressure (LVEDP) of 10 mmHg (Figure 3).

**Figure 4:**
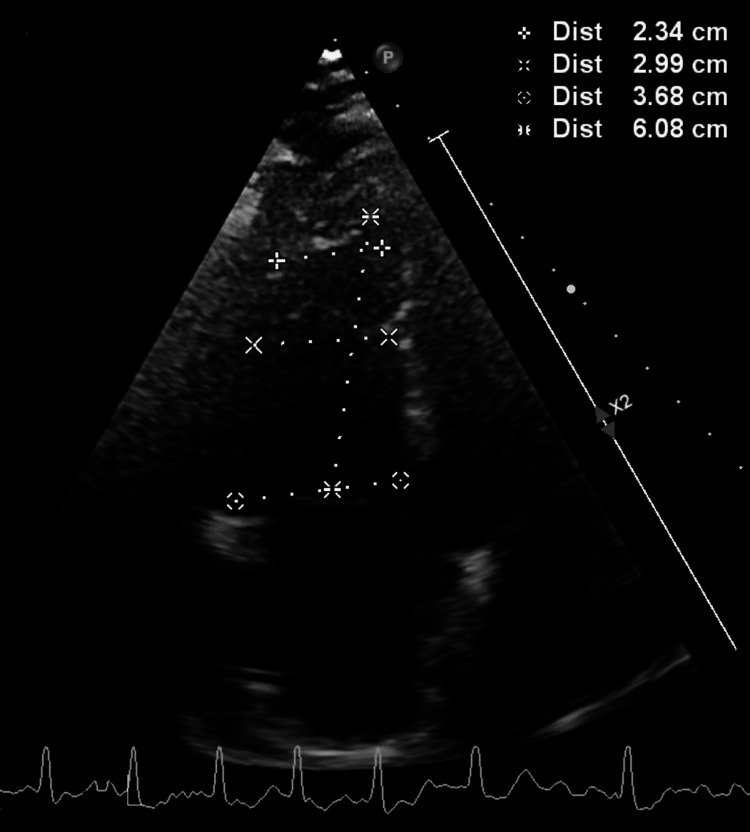
Transthoracic echocardiography (TTE) findings Apical four-chamber view showing a dilated right ventricle

The patient was admitted to the coronary care unit (CCU) for further care. A transthoracic echocardiogram (TTE) was obtained and showed normal left ventricular (LV) systolic function, an estimated LV ejection fraction (LVEF) of 55-60%, and hypokinesis of apex, anteroseptal, and inferoseptal wall segments (Video 1). The right ventricle (RV) was dilated and hypokinetic (Figure 4).

**Video 1:** Pertinent transthoracic echocardiography (TTE) findings Section 1 of the video: a parasternal long axis view showing hypokinesis of the anteroseptal wall (red arrow). Section 2: an apical four-chamber view showing hypokinesis of the apex and inferoseptal wall (yellow arrow)

**Figure 5:**
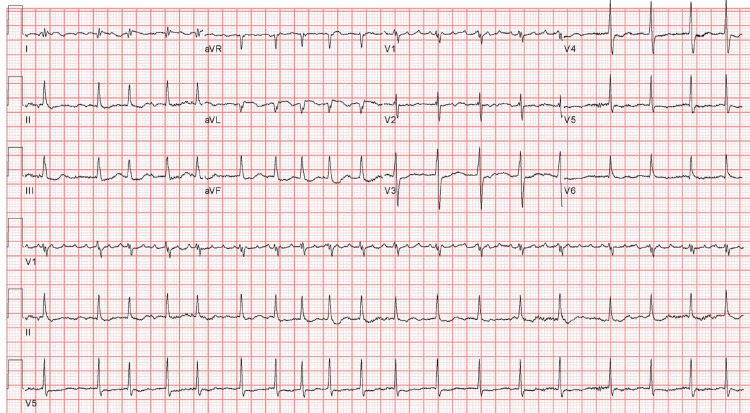
ECG ECG showing atrial fibrillation with a rapid ventricular response

While in the CCU, the patient developed atrial fibrillation with a rapid ventricular response (Figure 5), which was managed with a digoxin load of 1 mg, followed by a maintenance dose of 0.125 mg every other day as the patient could not tolerate beta-blocker therapy. In addition, the heparin drip and clopidogrel were continued, and aspirin was discontinued.

The patient was then transferred to the medical intensive care unit (MICU) for further workup of his acute hypoxic respiratory failure. Given the high suspicion of severe pneumonia due to his immunocompromise state, concern for aspiration due to a history of dysphagia, severe respiratory distress requiring HFNC, a relatively elevated white blood cell count of 9.8 K/mcL compared to his baseline of 4-5 K/mcL, and the bilateral ground glass airspace opacities on CTPA, he was started on IV cefepime 2 mg every eight hours, IV metronidazole 500 mg every eigth hours, and IV vancomycin 1500 mg loading dose. An extensive infectious and autoimmune workup for the non-specific ground glass opacities was negative (Table 2).

**Table 2:** Extensive infectious and autoimmune workup Ab: antibody, AG: antigen, ANA: anti-nuclear antibody, ANCA: antineutrophil cytoplasmic antibodies, CCP: cyclic citrullinated peptide, CMV: cytomegalovirus, GBM: glomerular basement membrane, Ig: immunoglobulin, MRSA: methicillin-resistant Staphylococcus aureus, PCR: polymerase chain reaction, Qual: qualitative, Quant: quantitative * indicates an abnormal value

Lab	Result	Reference range & units
Legionella AG, urine	Negative	Negative
Respiratory viral panel	Negative	Negative
Coccidioides Ab, IgM	0.1	Negative <1
Coccidioides Ab, IgG	0.3	Negative <1
(1,3) Beta-D-Glucan	36	< 80 pg/mL
Aspergillus AG	Not detected	Not detected
Galactomannan index value	<0.50	< 0.50
Histoplasma AG, urine	<0.2	Negative < 1.1 ng/mL
MRSA Nares DNA	Not detected	Not detected
Blastomyces AG, Quant	Not detected	Not detected
Cryptococcal AG, Qual	Negative	Negative
CMV Ab, IgG	1.70*	(0-0.59) U/mL
CMV DNA PCR, Quant	<137	<137 IU/mL
CMV DNA PCR, Qual	Not detected	Not detected
Lactate dehydrogenase	356*	(140-271) U/L
Sputum culture	Mixed normal oropharyngeal flora	
Complement C3	157	(87-200) mg/dL
Complement C4	70*	(19-52) mg/dL
CCP Ab IgG/IgA	6	(0-19) Units
Cytoplasmic Ab	<1:20	<1:20 Titer
Perinuclear Ab	<1:20	<1:20 Titer
Atypical P-ANCA	<1:20	<1:20 Titer
Anti-Myeloperoxidase Ab	<9	(0-9) U/mL
Anti-Proteinase-3 Ab	<3.5	(0-3.5) U/mL
ANA, Qual	Negative	Negative < 1:80
Rheumatoid factor	11	< 14 IU/mL
Anti-GBM Ab	4	(0-20) Units

Per infectious diseases (ID) specialists' recommendations, though low concern for pneumocystis jiroveci pneumonia (PJP), due to the patient's immunosuppression status and his respiratory condition, he was started empirically on IV trimethoprim-sulfamethoxazole (TMP-SMX) 15-20 mg/kg/day based on TMP content, in four equally divided doses for three days, which then switched later to oral primaquine 26.3 mg daily and IV clindamycin 900 mg every eight hours due to the volume overload associated with IV fluids given with TMP-SMX. In addition, a pulse steroid therapy of IV methylprednisolone 250 mg twice daily was initiated and continued for three days, followed by a tapering dose of oral prednisone. Furthermore, cefepime and metronidazole were switched to IV meropenem 500 mg every six hours to complete a total of a 14-day course for pneumonia. After seven days of PJP treatment, the ID specialists recommended discontinuing these antibiotics given the negative fungitell (1,3-Beta D Glucan) test and the lack of expected clinical improvement. The patient also received antifungal therapy of IV micafungin 100 mg daily and oral voriconazole 350 mg twice daily, which were discontinued after three days due to the negative fungitell test. In addition, the lack of neutropenia and imaging findings are not suggestive of pulmonary aspergillosis.

**Figure 6:**
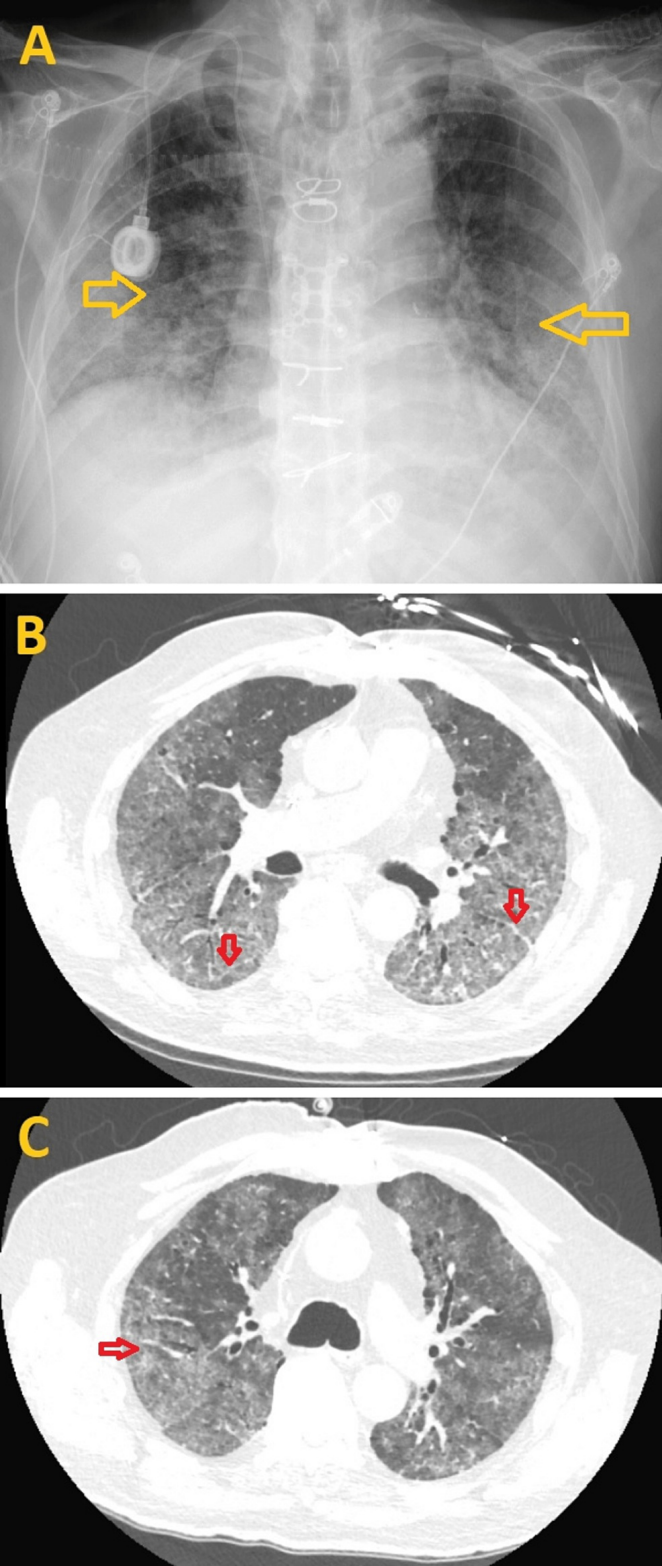
Repeat chest X-ray (CXR) and high-resolution chest CT scan findings 6A: CXR shows increased bilateral interstitial opacities involving the mid and lower zones of the lungs (yellow arrows). 6B, 6C: A high-resolution chest CT without contrast demonstrates stable bilateral ground glass opacities with septal thickening (red arrows).

During hospitalization, the patient had increasing oxygen requirements intermittently, requiring bilevel positive airway pressure (BIPAP) 14/5 with an average FIO2 of 70% and HFNC with an average FIO2 of 70% and flow rate of 50L/min, but he was never intubated. Due to his worsening respiratory status, a repeat CXR was obtained and showed increased bilateral interstitial opacities involving the mid and lower zones of the lungs (Figure 6A). A high-resolution chest CT without contrast revealed stable bilateral ground glass opacities with septal thickening (Figures 6B, 6C).

Sputum culture was repeated and grew *Stenotrophomonas maltophilia (S. maltophilia)* sensitive to minocycline, TMP-SMX, and levofloxacin, for which he received a 10-day course of IV minocycline 100 mg twice daily. The patient received intermittent IV Lasix throughout the hospital course for volume overload. Despite the antibiotics and steroid therapy, the patient remained on HFNC with no clinical improvement and was not better at any point during hospitalization to consider bronchoscopy. For weaning of his HFNC oxygen therapy, he was discharged to the long-term acute care hospital (LTAC), where he eventually passed away.

## Discussion

RTX-ILD is a rare but potentially fatal side effect of RTX therapy [1]. In a systemic review of 121 cases, RTX-ILD occurred predominantly in males and was most common during the fifth and sixth decades of life. The average number of cycles before disease onset is four, and the mean time from the last infusion to the disease onset or abnormal radiographic findings is 30 days. The most common presenting symptoms are dyspnea, fever, and cough. Radiographic abnormalities included bilateral interstitial infiltrates on CXR and ground glass opacification on high-resolution chest CT in 97.5% of the cases [2].

RTX-ILD can present in three forms with different underlying mechanisms. Early onset hyperacute form occurs within hours and is characterized by acute respiratory distress syndrome (ARDS). It is possibly related to infusion-related reactions, cytokine release, and tumor lysis syndrome, especially when the tumor burden is the greatest, as in patients receiving multiple cytotoxic agents and at the first cycle. Acute or subacute form occurs within days to three weeks, in particular organizing pneumonia, probably due to a hypersensitivity reaction to the chimeric anti-CD20 antibody. Lastly, Late onset or chronic may occur after several months and could be related to the drug toxicity or immune system restoration [1]

Our patient presented with worsening dyspnea, cough, and hypoxemia after the sixth cycle of RTX-bendamustine and after three weeks from the last infusion. The differential diagnosis of bilateral ground glass opacities in our patient includes community-acquired pneumonia (viral, bacterial, or fungal, including PJP), pulmonary edema, and RTX-ILD. The normal LVEDP during cardiac catheterization and the pattern of bilateral interstitial infiltrates on CXR make cardiogenic pulmonary edema less likely. The negative respiratory viral panel and extensive fungal infections workup exclude viral and fungal pneumonia. Furthermore, the extensive negative autoimmune workup excludes autoimmune diseases.

The rate of PJP is substantial in cancer patients, especially in patients with hematologic malignancies [3]. Although elevated LDH is used as an indicator of possible PJP infection in HIV patients, it is non-specific and can be elevated from underlying hematologic malignancy or other causes of acute lung injury, as in our case. Since the number of organisms is significantly lower in non-HIV patients compared to HIV patients, the definitive diagnosis of PJP requires identification of the organism either by polymerase chain reaction (PCR)-based assays, dye-based staining, or fluorescent antibody staining of respiratory specimens obtained preferably by bronchioalveolar lavage (BAL) [4]. Due to our patient's respiratory failure and the high oxygen requirement, bronchoscopy was deferred.

A meta-analysis of 13 studies demonstrated that a negative serum β-D-Glucan (BDG) is sufficient to exclude PJP in HIV patients; however, in non-HIV patients, clinical and radiological findings should be taken into consideration in parallel to the test [5]. We empirically treated our patient for PJP with TMP-SMX followed by clindamycin and atovaquone for a total duration of seven days in addition to steroid therapy; however, due to the low suspicion for PJP and the lack of clinical improvement, antibiotics were discontinued per the ID specialists' recommendations.

*S. maltophilia *is a multidrug-resistant opportunistic pathogen and is associated with high morbidity and mortality in severely immunocompromised patients. *S. maltophilia* pneumonia is usually hospital-acquired. Although it can colonize the upper airway and large bronchi without causing infection, *S. maltophilia* can present with rapidly progressive and fatal hemorrhagic pneumonia in patients with hematologic malignancies, so a positive sputum culture in a symptomatic immunocompromised patient should be interpreted as an infection, and not colonization [6]. Although *S. maltophilia *can present radiologically with ground glass opacities, as in our case, the first sputum culture was negative for *S. maltophilia*, and it is unlikely it was the cause of his ground glass opacities on presentation. Furthermore, we treated it with a 10-day course of minocycline with no clinical improvement.

The troponin T elevation up to 10 ng/mL and the ECG changes on presentation warranted a diagnostic cardiac catheterization, which revealed no new obstructive lesions and patent grafts. The differential diagnosis of elevated troponin in our patient is NSTEMI type I, NSTEMI type II/demand ischemia due to his severe respiratory illness, myocarditis, and Takotsubo syndrome.

Cardiac magnetic resonance (CMR) is an important tool to distinguish normal myocardium from Takotsubo and tissue scarring due to myocarditis or myocardial infarction [7]; however, given our patient's critical condition and worsening respiratory status, we could not obtain CMR. Furthermore, the echocardiographic findings of normal septal thickness, LVEDD, and LVEF and the absence of apical ballooning make myocarditis and Takotsubo less likely [8,9].

It is very likely that the patient's severe hypoxemia and the pulmonary process derived from his myocardial infarction and right ventricular dysfunction; however, due to the significant elevation of troponin levels more than expected for demand ischemia and taking into consideration his past cardiac history, we opted to treat the patient as NSTEMI I with dual antiplatelet therapy; aspirin and clopidogrel, which were later switched to clopidogrel and apixaban on discharge due to his atrial fibrillation [10].

Prognosis is variable in the literature. RTX-ILD can range from mild to rapidly progressive, fatal disease. Due to low suspicion or decreased awareness about RTX-ILD, the continuation of RTX therapy following the development of symptomatic disease is associated with worse outcomes [2]. Although many patients who had RTX-ILD achieved recovery with high-dose steroid therapy, it was unable to prevent death or mechanical ventilation in a number of patients, even in patients who received steroids as a part of the RTX regimen [1,2]. Hadjinicolaou et al. reported death in 18% (18/99) of patients with potential RTX-ILD [2]. In another systematic review, Lioté et al. reported a death rate of 24% (11/45) in cases with possible RTX-ILD [1]. 

Bendamustine is a cytotoxic alkylating agent that is used to treat indolent NHL, mantle cell lymphoma, and CLL. Myelosuppression is one of the common major side effects. Patients taking bendamustine are at an increased risk of PJP due to the decrease in CD4/CD8 ratio and suppression of the T cell-mediated immunity associated with the drug [11]. Bendamustine has not been linked to pulmonary toxicity in the literature. Only one case report suggested bendamustine as the causative agent of eosinophilic pneumonitis in a patient with stage 3A diffuse large B-cell lymphoma; however, he received six cycles of RTX as a part of R-CHOP combination therapy (RTX, cyclophosphamide, doxorubicin, vincristine, prednisone), followed by four weekly doses of RTX single-agent therapy [12].

## Conclusions

Rituximab is a well-established medication that is used to treat certain hematologic malignancies and autoimmune diseases. Although it is a relatively safe drug, it is associated with rare but potentially fatal pulmonary toxicity. Should patients on RTX develop new respiratory symptoms or radiological changes, RTX-ILD should be considered, and treatment should be pursued early as any delay is associated with worse outcomes.
